# Isolation of Circulating Tumor Cells by Dielectrophoresis

**DOI:** 10.3390/cancers6010545

**Published:** 2014-03-12

**Authors:** Peter R. C. Gascoyne, Sangjo Shim

**Affiliations:** 1 Department of Imaging Physics Research, The University of Texas M.D. Anderson Cancer Center Unit 951, 1515 Holcombe Boulevard, Houston, TX 77030, USA; 2 Department of Biomedical Engineering, The University of Texas at Austin, 1 University Station, C0800, Austin, TX 78712, USA

**Keywords:** circulating tumor cells, dielectrophoresis, field-flow fractionation, cell membrane, cell isolation, cell membrane, cell dielectric properties

## Abstract

Dielectrophoresis (DEP) is an electrokinetic method that allows intrinsic dielectric properties of suspended cells to be exploited for discrimination and separation. It has emerged as a promising method for isolating circulation tumor cells (CTCs) from blood. DEP-isolation of CTCs is independent of cell surface markers. Furthermore, isolated CTCs are viable and can be maintained in culture, suggesting that DEP methods should be more generally applicable than antibody-based approaches. The aim of this article is to review and synthesize for both oncologists and biomedical engineers interested in CTC isolation the pertinent characteristics of DEP and CTCs. The aim is to promote an understanding of the factors involved in realizing DEP-based instruments having both sufficient discrimination and throughput to allow routine analysis of CTCs in clinical practice. The article brings together: (a) the principles of DEP; (b) the biological basis for the dielectric differences between CTCs and blood cells; (c) why such differences are expected to be present for all types of tumors; and (d) instrumentation requirements to process 10 mL blood specimens in less than 1 h to enable routine clinical analysis. The force equilibrium method of dielectrophoretic field-flow fractionation (DEP-FFF) is shown to offer higher discrimination and throughput than earlier DEP trapping methods and to be applicable to clinical studies.

## 1. Introduction

Cellular characteristics that can be exploited as biomarkers of cancer are usually categorized according to whether they depend on molecular markers or cell physical properties [1,2,3,4,5]. The most common biomolecular target for the detection and isolation of circulating tumor cells (CTCs) from peripheral blood mononuclear cells (PBMNs) is EpCAM, a cell adhesion molecule present in most epithelial tissues, and methods of exploiting this marker through antibody labeling have played a crucial role in establishing the research and clinical significance of CTCs as prognostic and, potentially, diagnostic indicators for breast, prostate and other cancers [6,7,8]. EpCAM and several more specific antigens that have been identified as alternative markers for this purpose are not universally expressed by CTCs [9,10,11,12]. In addition, antibody-conjugation is time consuming and somewhat inefficient, and conjugation and subsequent cell recovery can impact CTC properties and viability. For these reasons there is interest in exploring the applicability of cell physical properties for identifying CTCs.

Physical markers have the advantage that they can be exploited not only as the means to discriminate between cancer and normal cells but also to create the forces that drive cell separation. Because this makes labeling of cells with antibodies or dyes unnecessary, cells isolated by exploiting their intrinsic physical properties remain unmodified and viable during separation. Physical characteristics that have been identified as markers for CTC isolation include cell size (through size-based filtration [13,14,15]), density (through density-gradient separation [16,17,18]), or both (through inertial-hydrodynamic discrimination [19,20,21,22]), as well as cell capacitance and conductivity (through dielectrophoresis (DEP) [3,23,24,25,26,27]). Both the size and density distributions of CTCs have been shown to overlap with those properties of peripheral blood mononuclear cells (PBMNs), resulting in inefficient separation of CTCs by size- and density-based methods [28,29] in some cases. Nevertheless, recent studies employing the NCI-60 panel of cancer cells suggest that cell membrane area characteristics exploited by DEP may be widely applicable to the isolation of CTCs derived from different cancer types [28,29,30,31].

In this article, the application of DEP to tumor cell detection and isolation will be discussed first with the aim of clarifying the biological basis of the cell dielectric differences that are used to discriminate between normal and cancer cells. Generally, the dielectric properties of cells are unfamiliar to oncologists and the goal here is to explain how these properties relate to more familiar cell structure-function relationships. Specifically, the dielectric differences between normal and cancer cells will be discussed in terms of the increase in cell membrane structure associated with cancer and differences between cells from solid tissues and blood in terms of cell structure and function relationships appropriate to these distinct sites of origin. From the principles presented it is inferred that the dielectric properties of CTCs shed from solid tumors, regardless of cancer type, will differ substantially from those of normal blood cells, suggesting that DEP-based methods should be applicable to the isolation of unmodified, viable CTCs from solid tumors of all types.

Having understood the basis for discriminating CTCs from blood cells, attention will be turned to the fact that CTCs are extremely rare in the peripheral blood of cancer patients and it is necessary to process blood specimens of 10 mL or more to isolate sufficient CTCs for meaningful analysis. Cell DEP is a microscale phenomenon and significant technical challenges arise in applying it in a manner capable of processing such huge numbers of cells with sufficient discrimination at high enough throughput to process clinical specimens in a reasonable time such as one hour. Unfortunately, articles proposing DEP approaches for CTC isolation continue to emerge that lack feasibility for scaling to clinical specimens. Commonly overlooked limitations are that cell concentrations must be kept sufficiently low to allow high discrimination in DEP and that cell properties can change over time through ion leakage as well as sensitivity to applied electric fields. To help clarify the needs, we have summarized the technical requirements of a practical DEP isolator for clinical applications based on our current understanding. We compare conventional DEP trapping and equilibrium force approaches for building DEP-based cell isolation devices that meet these requirements. A DEP-field-flow fractionation approach meets both the discrimination and throughput requirements and we point to recent findings showing successful, marker-free isolation of CTCs from clinical specimens using this continuous-flow DEP-FFF methodology.

By pulling together and reviewing these multifaceted aspects of cell dielectric properties, DEP principles, and practical design considerations for high throughput isolation for the first time within a single article, we hope to provide a useful resource for further improving DEP-based CTC isolation methods in the future. Our article is restricted to this focus and it does not pretend to be a comprehensive review of DEP or its other applications. Finally, in order to make this article as accessible as possible to readers from many backgrounds, we have relegated the supporting theoretical considerations to the appendix.

## 2. Dielectrophoretic Principles in the Separation of Cancer from Normal Cells

### 2.1. Dielectrophoresis

**Figure 1:**
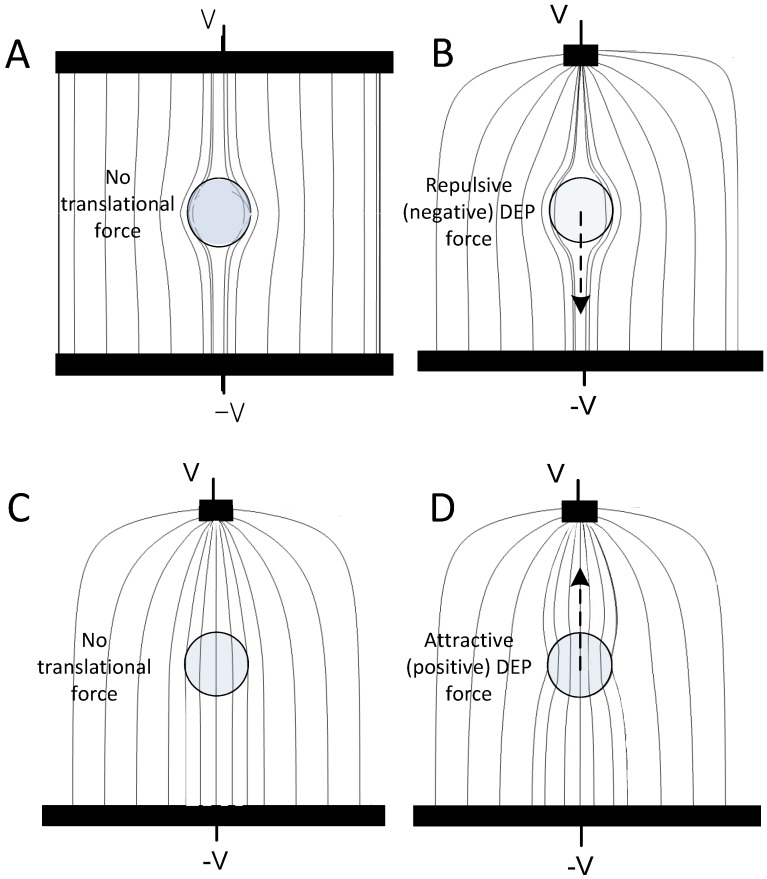
Deflection of electric field lines (gray lines) originating from electrodes (black bars) by mammalian cells. (**A**) In a low frequency electric field, an intact cell membrane accumulates charges that repel the field lines around the cell. If the field is homogeneous, then the perturbed field pattern will be symmetrical above and below the cell. No net force on the cell results; (**B**) If the electrode system imposes an inhomogeneous electric field, then the displacement of field lines is asymmetrical above and below the cell. This leads to a spatial energy gradient and a dielectrophoretic (DEP) force that pushes cells away from the high field region where the field lines are close together; (**C**) If the cell membrane is leaky and presents no barrier to the field, or if the applied field is at the cell crossover frequency, or if the field frequency is very high and the cell interior conductivity matches that of the suspending medium, then the field lines are not perturbed and the cell experiences no *F_DEP_* even in an inhomogeneous field; (**D**) At high frequencies, field lines are deflected towards the cell interior if the cell internal conductivity exceeds that of the suspending medium. In this case, the resultant energy gradient provides an *F_DEP_* that pulls the cell towards high field regions.

Dielectrophoresis is the movement of particles caused by asymmetrical displacement of electric fields. When an alternating electric field is applied to a suspension of viable cells, the cell membrane exteriors accumulate surface charges from the ionic medium that tend to repel electric field lines so that ion currents flow around the cells [32] (see Figure 1A,B). The time taken after the field is switched on for ions in the suspending medium to charge up the cell membrane and repel the field lines depends on the ion concentration of the suspending medium (*i.e.*, its conductivity) and the area of cell membrane that must be charged up. If the field is not static but oscillates at low frequency, the membrane charging will follow the field reversals. The repulsion of the field lines around the cells represents a higher energy state than if no cells were present in the suspension. If the applied field is homogeneous then the deflection of field lines and resultant perturbations of electric energy densities around each cell are symmetrical. The total energy of the system changes but the symmetry of the perturbation causes there to be no net force tending to move the cell (Figure 1A). However, if the field is inhomogeneous, the deflection of field lines and resultant perturbations of electric energy densities around each cell are asymmetrical. The total energy of the system becomes a function of cell position and a net force, called the dielectrophoretic force, arises (Figure 1B). When the field lines are excluded from the cell, the DEP force *F_DEP_* tends to displace the cell away high field regions (Figure 1B).

If the field frequency is increased, ions in the suspending medium will no longer have enough time to fully charge up the cell membrane exterior at each field reversal. As a result, the deflection of the field caused by the charge build up is less than maximal. At extremely high frequencies, there is essentially no time for ions to charge the outside of the membrane at all. If the ionic conditions inside and outside the cells are similar, the field lines will then pass undeflected into the cells (Figure 1C) at such high frequencies and the cells are essentially indistinguishable from the suspending medium from a dielectric standpoint because no deflection of the electric field occurs. In this case there is no *F_DEP_*.

Finally, if the suspending medium is of low conductivity and the cell interiors are of high conductivity, cell interior ions will have time to build up on the inside of the cell membrane even though ion build-up on the outside of the membrane is incomplete. In this case, electric field lines will be attracted towards the cell interior and the field will become more concentrated inside the cell than on the exterior (Figure 1D). This perturbation, in which field lines are concentrated rather than repelled, leads to a lower overall electric energy than if no cells were present in the suspension. In an inhomogeneous electric field, this perturbation leads to a positive *F_DEP_* that attracts cells towards high field regions.

Unlike electrophoresis, DEP does not depend on net charges being affixed to the cells and it occurs only in inhomogeneous electric fields. Significantly, the direction of *F_DEP_* is determined not by the direction of the electric field but by the direction of the field gradient defined by asymmetry in the system that generates the field. Most significantly, this independence of on field direction allows alternating electric fields to be used to manipulate cells and permits different cell types to be discriminated on the basis of their frequency-dependent dielectric properties [32] and independently of their net surface charge.

It follows that viable cells suspended in a sufficiently low conductivity medium will experience an *F_DEP_* in an alternating inhomogeneous electric field that will push them away from high field regions when the field frequency is low (negative DEP, Figure 1B) and will pull them towards high field regions when the field frequency is high (positive DEP, Figure 1D). As the frequency traverses a well-defined intermediate “*DEP crossover frequency*”, *F_DEP_* passes through zero and changes direction (Figure 1C).

Different cell types having different surface area and size characteristics exhibit different DEP frequency responses and it is possible to choose an electric field frequency that lies in between the crossover frequencies of different cell types. In this case, cells with the lower crossover frequency will be attracted towards high field regions (e.g., electrode edges or pinched field regions) while cells of higher crossover frequency will be repelled towards low field regions. In this way, DEP may be used to discriminate between different cell types because DEP crossover frequency depends on the ionic conductivity of the suspending medium, which can be adjusted, and on the cell size and surface area. The DEP crossover frequency is the essential parameter that is exploited for separating cells through the choice of appropriate experimental conditions especially the electric field frequency of the applied DEP field. To achieve sorting, cells flow through a thin chamber that has a means of creating an inhomogeneous electric field, such as an array of microelectrodes or an array of dielectric posts with accompanying current-driving electrodes. Cells will be repelled from the high field regions on the array if the applied field frequency is lower than their crossover frequency and attracted to the high field regions if the applied field frequency is higher than their crossover frequency. DEP isolation of different cell types relies on counter-motion of the different cell types in response to the DEP field. Because isolation of different cell types depends on cell crossover frequency differences, we will examine in some detail the origin of the cell dielectric properties that underlie this parameter.

For those interested in a more formal analysis for *F_DEP_* in terms of the electric field inhomogeneity and the cell crossover frequency characteristics, these may be found summarized in Equations (A1)–(A3) in the Appendix.

### 2.2. Cell Membrane Dielectric Properties

Pohl first demonstrated the manipulation of cells by DEP and this and the related electrokinetic method of electrorotation were subsequently applied to characterize the dielectric properties of cells and to manipulate and isolate different cell types from mixtures [32,33]. These methods easily distinguished between viable and non-viable cells [34] and were used to detect differences between different cell types [35,36,37,38] and observe responses of cells to many types of stimuli including exposure to toxicants [39,40,41], viral [42,43] and parasitic [44,45,46] infections, and treatment with cell differentiating- [47,48,49,50,51], mitogenic- [52], and apoptosis-inducing- [41,53,54,55] agents, for example. Electrokinetic methods were honed as important tools for analyzing cell physical properties [56,57,58,59]. Excellent reviews covering these topics and their application to many cell types have been provided [60,61,62,63]. In general, these studies showed that DEP and ROT could sensitively detect changes in membrane area or membrane conductivity resulting from stimuli, challenges or physiological changes. In experiments relating to cancer, consistent dielectric differences have been found between cells whose phenotypes can be manipulated between normal and transformed states by physical, chemical and molecular agents [47,49,64,65]. We demonstrated that the basis for cell DEP crossover frequency differences lay in cell membrane morphology [49,51].

The cell plasma membrane of most cell types is not smooth but contains both small and large cell surface features including microvilli, folds and ruffles that cause mammalian cells to have larger membrane surface areas than idealized, smooth spheres of similar volume [49,51]. To quantify how the total surface area of a cell exceeds the area of a perfectly smooth body of similar volume, we introduced the concept of a membrane folding factor, *ϕ*, the ratio of actual cell membrane area to the area of a perfectly smooth cell of similar volume [51].

**Figure 2:**
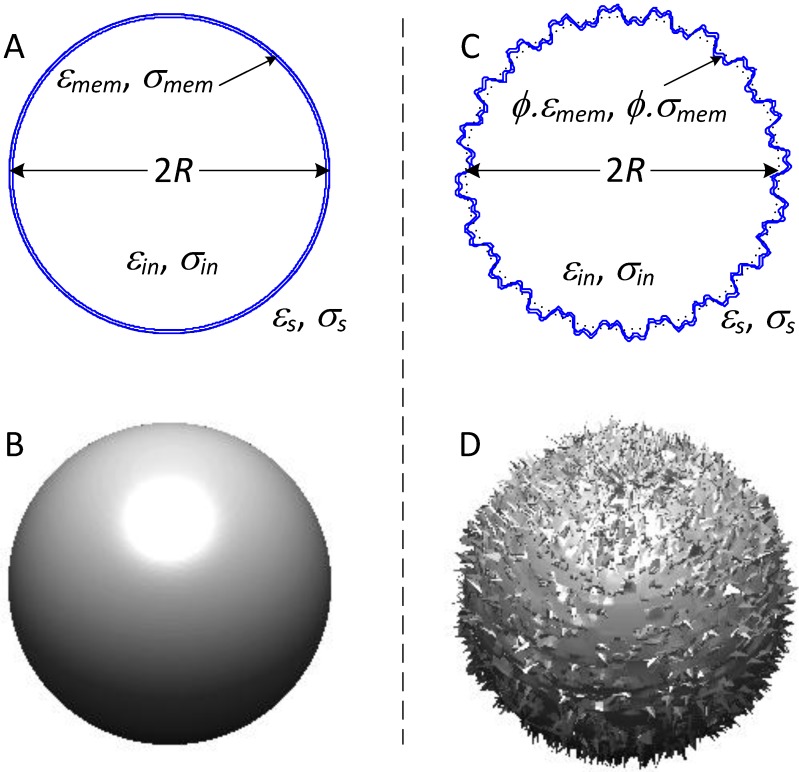
The DEP responses of mammalian cells may be understood in terms of dielectric shell models [66,67,68]. In (**A**) the cell is represented as a homogeneous core (the cytoplasm) surrounded by a thin, homogeneous shell (the lipid bilayer membrane). This model describes the perfectly smooth, idealized cell shown in (**B**); In reality, mammalian cells have surface morphological features such as those represented in (**D**) that are covered by lipid bilayer membrane and that increase the cell surface area compared to the smooth idealized cell; (**C**) These morphological features can be taken into account by introducing a folding factor *ϕ* into the shell model to represents the ratio of actual lipid bilayer area to that of the idealized smooth shell. Symbols: *ε* and *σ* refer to the real permittivity and conductivity, respectfully, of the cell components denoted by the subscripts *s*, *mem* and *in*, which refer to the suspending medium, plasma membrane and cell interior, respectively. *ϕ* is the membrane folding factor (see text).

Mammalian cell dielectric properties may be derived theoretically using a single shell model (see Figure 2) to approximate the electrical characteristics of the salient cell structures. Cell morphology need not adhere exactly to the idealized forms shown in Figure 2 in order for the shell model to provide a reasonable approximation of the cell dielectric properties. The overall DEP properties of a cell in suspension derive from the volume integral of the perturbation in the overall field distribution around the cell, which encompasses a very much larger volume than the cell itself (see Figure 1). While the overall field perturbation depends upon the total charge capacity of the cell plasma membrane (which is proportional to membrane area), morphological imperfections at the cell surface play only a minor role in shaping the overall field perturbation and in determining the consequential dielectric and DEP properties. Even the dielectric properties of fairly irregular cells such as those shown in Figure 4F seem to be described surprisingly well by a spherical single shell model that incorporates membrane folding.

The formal relationship between the cell size, membrane folding factor and the DEP crossover frequency are derived in the appendix. For cells having an intact membrane barrier function in a medium of much lower conductivity than their cytoplasm, the DEP crossover frequency is shown (Equations (A4)–(A9)) to depend on the product of the cell radius *R*, the membrane folding factor *ϕ* and the capacitance per unit area of smooth membrane *C*_0_. Indeed, (*R*∙*ϕ*) defines the “*dielectric phenotype*” of a given cell type and determines how it will respond to DEP manipulation. This sensitive dependency of the dielectric phenotype on membrane folding, in addition to cell size, distinguishes DEP methods from approaches to cell isolation that depend on size alone, such as size filtration.

### 2.3. Cancer Cell Dielectric Properties, Tissue Morphology and Cancer Progression

**Figure 3:**
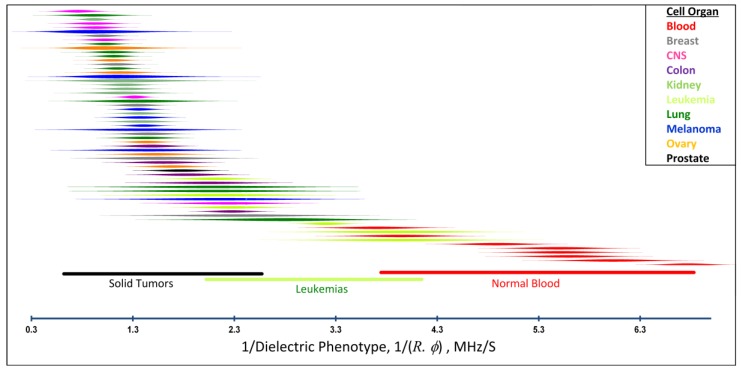
The DEP responses of cancer and normal blood cells expressed in terms of the reciprocal cell dielectric phenotype 1/(*R*∙*ϕ*), which is proportional to the DEP crossover frequency that determines the behavior of the cells in DEP manipulation and isolation applications. Each line shows the distribution of crossover frequencies among cells of a single cell type. Cell types are color-coded by organ of origin. The results show that there are striking differences between the DEP properties of blood cells and cells of solid tumor origin and that leukemia cells display intermediate properties. More details of these data, including a description of the cell type names, origin and methods of measurement have been published elsewhere [28,29].

Over a number of years a large number of cancer cell types of both cultured and primary origin have been examined and a consistent trend of cancer cells having larger folding factors and radii than both normal cells of comparable origin and blood cells has emerged [26,36,64,69,70,71,72,73,74,75,76,77,78,79]. Most recently we published a DEP characterization of the NCI-60 panel of cancer cell types and showed that all of the cell lines derived from solid tumors have crossover frequencies that should allow their efficient isolation from normal blood cell types [29]. We have explicitly demonstrated the isolation of several of these cell lines from blood as examples [23,28,78,79,80]. A visual summary of those results for tumor types of different organ derivations is shown in Figure 3.

The cells of transformed phenotype tended to exhibit more surface features, typically giving them a 50% to 300% larger capacitance per unit area than cells of normal phenotype. This membrane area effect was shown to far outweigh other possible influences on cell membrane capacitance in cancer versus normal cells such as changes in protein/lipid composition in the membrane and membrane thickness [51]. In addition to having greater membrane folding factors *ϕ* than normal cells, cancer cells also tended to have larger radii *R* than their normal counterparts [51]; both factors contributed to differences in cell dielectric phenotypes (R∙*ϕ*) between transformed and normal cells.

The combined folding factors and sizes of leukemia lines were also found to be distinct from normal blood cells but the differences were smaller than for the solid tumor types. These differences between leukemia and blood cells would allow leukemia cells to be concentrated from blood by DEP methods but with less efficiency than can be expected for cells of solid tumor origin [79,81].

Notwithstanding these experimental findings, without there being a sound biological basis for understanding the observed dielectric differences between cancer cells and blood cells, it is reasonable to question how widely applicable DEP might be to the isolation of CTCs originating from uncharacterized tumor types. To probe this question further, we investigated forty types of cultured cells prior to harvest in order to explore the relationship between their morphological properties during growth and their dielectric properties when they were released into suspension [29]. We showed that the cell membrane properties of the cells in suspension depended on their morphology at their growth sites prior to harvest. When a cell is an integral part of a tissue, it adopts a membrane area and volume that conforms to the cells surrounding it. However, once a cell loses anchorages and is released into suspension, cell cytoskeletal tension causes it to round up into a pseudo-spherical shape. As long as the cell remains intact, the cytoplasmic volume and the cell membrane area are both conserved during this process. Therefore, the membrane area, which was large enough to conform to neighboring cells in the tissue, becomes draped around the pseudo-spherical cell. Any filopodia and microvilli that existed in gaps between cells in the source tissue add further to the overall membrane area.

Figure 3, which shows the single shell dielectric model that is widely applied for the analysis of the dielectric properties of mammalian cells, modified to incorporate this membrane folding concept through undulations in the membrane surface. In practice, this model is adequate to describe CTCs that are not perfectly spherical as well as those with more highly-folded surfaces than depicted. Expressions summarizing the relationships between cell morphology in the tissue of origin, the membrane folding factor, and DEP properties of cells in suspension are given in the Appendix in Equations (A10)–(A13). Significantly, the analysis reveals that the dielectric phenotype of the cell in suspension can be inferred from its morphological characteristics in the tissue of origin. Specifically, the DEP crossover frequency once a cell enters suspension is expected to be proportional to 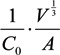, where *V* is the volume of the cell and *A* is its total surface area at its site of origin no matter what shape it assumes in the solid tissue. In principle, *V* and *A* can be estimated by examination of the tissue. This relationship provides, for the first time, a means to translate the morphology of cells in solid tissue as viewed by a pathologist to the DEP characteristics those cells will exhibit when they are released as CTCs.

**Figure 4:**
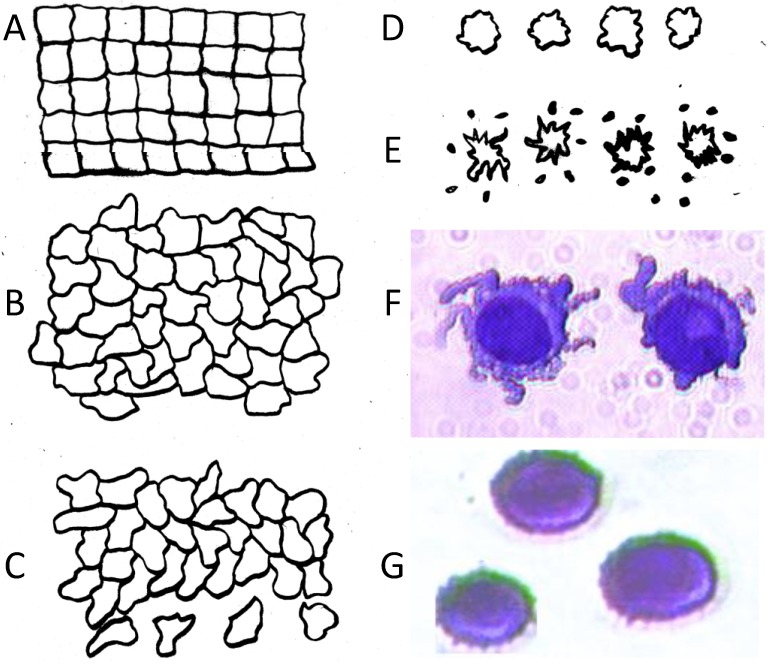
Morphological aspects of circulating tumor cells derived from solid tissues illustrated in 2D. (**A**) The minimum surface area cells can possess when they form solid tissue occurs when in a uniform stacked conformation. In this arrangement, the surface area of the cells exceeds that of smooth spherical cells of the same volume by a factor *ϕ_mn_* = 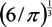 ≈ 1.24; (**B**) Tumor cells become disorganized as tumor grade increases, leading to increased cell surface area; (**C**) As tumor cells enter the circulation and round up into spheres, their surfaces wrinkle to accommodate their membrane area (**D**) leading to a low DEP crossover frequency; (**E**) Cell membrane area is shed in large vesicles when cancer cells persist in suspension [78]. Nevertheless, the membrane folding factor of the cells still remains high; (**F**) MDA-MB-231 cells in suspension immediately following harvest showing excess membrane and gross folding. (**G**) The same cells after being maintained in suspension for 2 h. Despite the loss of large vesicles carrying away cytoplasm and membrane, the cell membranes retain a much higher folding factor than blood cells [78].

While cells in suspension have a minimum surface area if they are smooth spheres, cells comprising solid tissues have a minimum possible surface area when they are built into a lattice resembling a 3-D stack of cubic bricks (see Figure 4). In this configuration, approximated in real life by well-ordered epithelial layers, cells have a membrane area that is larger than spherical cells of the same volume by a factor *ϕ_mn_* = 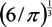 ≈ 1.24. This value, then, is the minimum folding factor possible for cells from well-differentiated, solid tissues. Cells that are not smooth and originate from less well organized tissues will therefore have folding factors, when harvested, in excess of 1.24. In practice we have found from DEP and ROT measurements on cells harvested from tissues that folding factors *ϕ* can range up to and even above 10. For example [82], perfusion by trypsin solution of the livers of euthanized mice via the hepatic portal vein yielded a harvest of hepatocytes ranging from diploid to 16-ploid having diameters from 15–40 µm and membrane folding factors from 4 to 10.5.

To understand why tumor cells tend to have larger membrane areas than normal cells, it is useful to consider that cancer is an entropic disease on every level of organization [83,84,85]. As it progresses, genomic [86], chromosomal [87,88,89], nuclear and cell internal structure [90,91,92] and gross cell [93,94,95] and tissue [85,96,97] morphology become increasingly disorganized until, in its later stages, tumor cells invade neighboring tissues and take flight throughout the body as circulating tumor cells that disseminate the tumor and eventually degrade even gross organismal structure. The primary measure used by pathologists to quantify how far cancer has progressed is tumor grade, defined as the degree to which cell morphology has altered and the structural organization of the tissue has broken down compared with normal, well-differentiated tissue. As cancer progresses and tumor grade increases, the area of membrane needed for cells to conform to their more disorganized neighbors increases [98,99]. Amplifying this effect is the concurrent degradation of inter-cellular adhesion complexes, including adherens junctions [100], tight junctions [101,102], connexin expression [103] and gap junctions [104], which normally retain close contact between the membranes of neighboring cells in normal tissue and eliminate gaps. As inter-cellular adhesion degrades, expression of membrane-rich filopodia [105] and invadopodia [106,107,108] occurs in gaps that form between neighboring cells. Some cells develop the ability to slither past their neighbors and extravate [108,109] into surrounding normal tissues. This motility, which itself is enhanced by the development of filopodia and invadopodia [108], leads to proximal metastasis and to the passage of tumor cells through blood vessel walls into the circulation to become CTCs.

It follows that increases in cell membrane area not only accompany the steps of progression but also play an essential roles in the dysfunctional behaviors of the cancer cells that eventually lead to CTC formation. From this established knowledge, increased cancer cell membrane area is seen to be a physical attribute of progression and is expected to reflect tumor grade. It is not surprising therefore that we and others have observed increased specific membrane capacitance values in cells from comparable transformed versus normal tissues [48,49]. These principles apply to any type of solid tumor that becomes invasive, suggesting that cell dielectric phenotype, which may be exploited through dielectric measurements or DEP manipulation, should be a widely applicable physical attribute of cancer cells and CTCs. Recently, the dielectric properties of mammalian cells have been linked to specific gene pathways involved in cell transformation and future molecular studies should provide additional understanding of the links between cell transformation and morphology that we have shown here to underlie DEP isolation. Specifically, Memmel *et al*. demonstrated that the surface morphology and size of glioblastoma cells, the parameters that determine cell dielectric phenotype and thereby facilitate DEP isolation, was strongly impacted by deregulation of the PI3K-AKT-mTOR pathway through modulation of PTEN and p53 expression [110]. Perturbations of this pathway have been implicated in the development of a cancer phenotype characterized by high glycolytic metabolism, increased cell size, and poorly regulated cell replication. It would not be surprising, therefore, if correlations are found between cell dielectric phenotype and other cancer properties that increased membrane area would facilitate such as enhanced glycolysis and the rapid glucose uptake detectable by PET imaging, for example.

### 2.4. Distributions of Cancer and Blood Cell Dielectric Properties and Isolation Efficiencies

We have seen that suspended cancer cells have dielectric properties that may be expected to depend on the grade of their tumor of origin. To date, we have examined more than 80 tumor cell types (data compiled from [28,29,47,48,49,50,51,52,65,78,79,81,111,112,113,114,115] and unpublished data) for which the mean folding factor was *ϕ_cancer_* = 2.2 ± 0.44. The mean crossover frequencies for these different tumor types ranged from 20 kHz ≤ *f_0cancer_* ≤ 75 kHz at 30 mS·m^−1^. In contrast, peripheral blood cells do not conform to neighboring cells to compose solid tissue, they have small sizes that enable them to travel through microcapillary beds, and they exist in a nutrient-rich environment where their metabolic exchange requirements can be met by transport across minimal membrane surface area. Accordingly, their membrane folding factors are closer to the value for smooth spheres, *ϕ* = 1.00. For the 15 subpopulations of normal peripheral blood cells we have examined, the mean folding factor was found to be 1.36 ± 0.25 and DEP crossover frequencies for all peripheral blood cell types exceed 120 kHz at a suspension conductivity of 30 mS·m^−1^. For five leukemias, we found an intermediate mean folding factor of 1.43 ± 0.30 and crossover frequencies between 60 kHz and 100 kHz at 30 mS·m^−1^.

Interestingly, if cells that normally grow in contact with one another are harvested and maintained in suspension for an extended period of time, they tend to undergo cytoplasmic remodeling whereby excess membrane and cytoplasm are shed via large vesicles [78]. This process, which is unrelated to apoptosis or loss of cell viability, results in a reduction of cell size but the cell membrane folding factor still remains high [78] and the cancer cells maintain a dielectric phenotype that is distinct from blood cell types that allows them to be isolated. This is significant because it suggests that it should be possible to use DEP to isolate circulating tumor cells from blood even if they exhibit similar membrane and cytoplasmic shedding in circulation and have thereby attained similar sizes to blood cells after they leave their tumors of origin.

## 3. Practical Systems for Separation of Cancer and Normal Cells for Clinical Applications

### 3.1. Summary of Requirements for DEP Implementations

The foregoing sections provide a background to DEP and an understanding of the dielectric differences between normal and cancerous cells and these help define practical requirements for DEP-based systems for isolating CTCs. Additional requirements include achieving sufficient throughput to provide for cancer cell isolations in a reasonable amount of time for routine measurements and maintaining the health of the isolated cells. These requirements define the basis for the design of practical DEP isolators no matter whether an electrode- or an electrodeless-based configuration is chosen.

Our early studies demonstrated proof of principle for isolating cancer cells from blood using microfluidic devices that employed DEP trapping—the retention of cancer cells in high field regions at electrode edges while blood cells were repelled and flowed to waste [79,80,81]. Since then various other approaches to cell isolation employing DEP have been presented. In general, two broad categories of DEP methods have emerged. In the first category, a positive *F_DEP_* is used to trap target cells in a potential energy well and to retain them until they are released by changing the DEP signal or fluid suspension conditions. These can be considered to be non-equilibrium methods because target cells are retained by a *F_DEP_* that exceeds the drag forces from fluid flow across the potential barrier at the same time as non-target cells remain untrapped and are eluted by fluid flow. In the second category, target and non-target cells are differentially positioned in the fluid flow inside the isolation chamber through balances of DEP and other forces and the cells move through the isolation chamber along different paths in accordance with their different physical properties. Target and non-target cells emerge either from a single exit port at different times or through different ports. Methods in the second category can be considered to be equilibrium methods because of the continuous force balances that are experienced by both target and non-target cell types during transit through the isolation chamber. The pros and cons of these approaches will now be considered.

### 3.2. Non-Equilibrium Isolation Approaches—DEP Trapping and Deflection by Potential Barriers

It became apparent during our early studies that, despite demonstrating the applicability of DEP to cancer cell isolation applications, DEP trapping had several shortcomings. The first limitation of DEP trapping approaches, which include not only the immobilization of cells in high field regions but also the shepherding of cells laterally in an energy well along the edge of an electrode into target- and waste-cell paths in a flow stream, is the tendency for cells to jump over the potential barrier that constrains them as a result of random fluctuations (Brownian motion). This is especially problematical when the cells are restrained by only one, or a limited number, of potential wells and the time for which they must be restrained is long (hundreds or thousands of seconds). By examining the behavior of cells in a chamber lined with an extended microelectrode array capable of trapping cells over and over again in the presence of fluid flow, we showed that, in reality, cells that were thought to be in a stable DEP trapping regime slowly “hopped” downstream from one trapping site to another and, given long enough, the cells always emerged from the DEP traps no matter what conditions were used [56]. Therefore, the concept of DEP “trapping”, which we and others had once used extensively, turns out to be valid only on a time scale that is short compared with the mean time taken for cells to escape from the trap. Therefore, the effective trapping efficiency falls continuously with time and drops precipitously on the timescale of the mean escape time, which depends on *F_DEP_* provided by the trap as well as the fluid flow rate. If it is not necessary to release the cells, the efficiency of a trap based on positive DEP attraction can be improved by anchoring the cells via adhesion molecules after are pulled into the trap [112]. If it is desired to release the cells later, then instead of using a single trap, a large number of opportunities for re-trapping the cells may be provided. When cells are to be constrained behind a potential barrier created by DEP repulsion, multiple opportunities for re-trapping the cells may also be provided. Then the overall behavior of cells of the same type to pass through a maze of traps will tend to average out over multiple trapping events. Important design aspects of a device for separating cells by DEP trapping, then, are the number of opportunities each cell has to be re-trapped as it flows through the device as well as the length of time the target cells need to be restrained or the distance they need to be deflected to isolate them from other cell types in the starting mixture. Recent approaches to DEP sorting using discrete potential barriers combine hundreds or thousands of trapping or deflection sites so that the stochastic characteristics average out as the cells undergo multiple encounters on their path through the DEP device [116,117,118].

Of course, the overall efficiency of cell restraint in a potential well is strongly governed by the force provided by the DEP trap [119]. When it is desired to discriminate between different cell types, this is determined not only by the device design and its operating parameters but also by the difference in the dielectric properties of the cell types to be separated. If different cell types have DEP crossover frequencies that are close together in value, then the maximum *F_DEP_* that can be applied to constrain one cell type while leaving another free is small. Some time ago we defined a “separability” parameter that predicts how readily one cell type may be separated from another by DEP restraint. The separability parameter depends on the differences between the cell membrane folding factors and sizes of the target and non-target cells to be discriminated. In other words, separability depends on the cell dielectric phenotype described earlier and, by extension, to differences in cell morphologies at the site of origin of the cells in the specimen. The separability falls towards zero if there is little or no difference between the dielectric phenotypes of the cells to be sorted (these relationships are considered theoretically in the Appendix).

The time for which target cells can be restrained becomes an all important consideration in practical DEP separation applications because a low retention time for target cells makes it impossible to sort the large numbers of cells in a large specimen over an extended period of time. The relationship between cell dielectric properties and DEP trapping times is treated theoretically in the appendix. In practice, the mean escape time for a cell after it has been trapped falls precipitously as the DEP trapping force, again determined by cell morphologies via the separability parameter, decreases.

As shown earlier, in order to exhibit positive DEP, the conductivity of the cell cytoplasm must greatly exceed that of the suspending medium. This can be achieved by suspending the viable cells in a low conductivity buffer prior to DEP processing. However this introduces a new time dependency into the cell separation efficiency considerations because cells slowly leak cytoplasmic ions once they are placed in a low conductivity medium. As a result, their DEP properties change on a time scale of around 1,000 s, setting an upper practical time limit for DEP discrimination and trapping [23]. Because *F_DEP_* increases with the square of the electric field, DEP traps are normally operated at as high a voltage as possible to increase the achievable trapping force and retention time. However, in the higher electric fields induced by a larger DEP voltage, the cells will not only experience a greater trapping force but also may exhibit field-enhanced ion leakage (or at even higher voltages electroporation and, in really extreme cases, electrodestruction [66,120,121]). The resulting rapid loss of cytoplasmic conductivity will *reduce* the cell trapping time. This effect is especially important for large cells because the transmembrane voltage induced by the DEP field, which is instrumental in inducing the ion leakage, is proportional to cell size (induced membrane voltage *V_membrane_* ∝ *R*.*E_local_* where *E_local_* is the local electric field acting across the cell).

A final important factor that can impact the efficiency and purity when separating cells by DEP is the cell loading concentration. As illustrated in Figure 1, the *F_DEP_* experienced by a cell depends on the extent to which the cell deflects the electric field. If another cell is nearby, mutual field perturbations will modify the *F_DEP_* experienced by both cells. This effect, which is generally referred to as an electric dipole-dipole [122,123] interaction, can result in the clustering of cells and the entrapment of both similar and dissimilar cell types [23]. Excessive cell loading was shown to lead to reductions in cell discrimination and separation efficiency in DEP. For example, on an interdigitated array of 50 µm electrodes with 50 µm gaps, the isolation efficiency was 90% at a loading density of 500 peripheral blood cells per mm^2^ but only 20% at a loading density of 10,000 peripheral blood cells per mm^2^ [23]. Therefore, to avoid problems caused by dipole-dipole interactions, cell loading must be limited so that the average cell spacing during DEP sorting is three cell diameters or more. Criteria for achieving this in DEP separation devices have been presented in detail elsewhere [31]. In CTC applications, this limits the maximum concentration of cells that can be resident in the DEP device at any time. The product of this maximum concentration and the flow rate then determines the cell throughput capabilities of a given DEP separator design. The number of target CTCs in clinical specimens is very small and interactions between CTCs do not impact device performance; rather it is overloading of the separator with PBMNs that impacts performance. This is important because it means that DEP isolation efficiency of CTCs is independent of CTC concentration.

### 3.3. Equilibrium Isolation Methods—DEP-FFF

To overcome limitations of DEP trapping and allow the isolation of cell types having very small differences in their DEP crossover frequencies to be achieved, we combined DEP with field-flow fractionation to create DEP-FFF, an approach in which tiny differences in the heights to which cells are repelled by DEP over a periodic microelectrode array are translated into differences in transit velocities through a chamber lined with DEP electrodes [124,125]. A detailed review of the development of DEP-FFF and the labs that contributed to its success has been provided recently [126] and the emphasis here will be on aspects that impact cell discriminating ability and throughput. Different cell types starting off together as a batch at the front of the separation chamber are continuously subjected to a combination of DEP, sedimentation and hydrodynamic forces and move to equilibrium heights at which these forces balance. Once again, the *F_DEP_* experienced by the cells is determined by the cell dielectric phenotypes that derive from the cell morphological characteristics discussed earlier. The equilibrium heights attained by the cells at force equilibrium, in turn, determine the velocities with which the cells are carried through the separation chamber by the parabolic flow profile of the eluate stream [124,125]. Because of Stokes drag, cells take several seconds to move to equilibrium heights in the chamber and this slow time constant has the beneficial consequence of averaging out the random competing fluctuations of Brownian motion during cell transit through the DEP-FFF chamber. Because thermal disturbances to cell height in the chamber are minimized, even cells having tiny dielectric differences average slightly different transit speeds in DEP-FFF and, if the chamber is long enough, the tiny resultant differences in cell velocities bring about complete separation of even subtly different cell types. These operational characteristics allow cells to be characterized and isolated with much higher discrimination by DEP-FFF than can be achieved by non-equilibrium DEP methods such as trapping and shepherding along potential barriers or by single electrode element DEP deflection methods that are sensitive to the initial positions or trajectories of the cells. Batch mode DEP-FFF has allowed us to characterize the statistical distributions of the dielectric properties, densities, and hydrodynamic properties of many types of cancer cells (see, for example, reference [56]) and paved the way for understanding the relationships between cell dielectric properties and morphology at the growth site of origin as described above [29]. Furthermore, construction of the microelectrode arrays using conventional, vendor-provided circuit board fabrication services and simple lamination techniques rather than cleanroom-based micromachining methods renders construction of high resolution DEP-FFF devices straightforward, inexpensive and readily accessible [127,128].

When applied to the problem of CTC isolation, batch mode DEP-FFF allowed us to separate cancer cells from PBMNs with over 90% efficiency [31]. Unfortunately, we found that even a relatively large DEP chamber 25 mm wide and 300 mm long could separate a batch of only a million or so cells every 20 min because, as already discussed, cell loading density must be kept low enough to avoid cell-cell dielectric interactions [31,122]. Because of this issue, batch sizes must be limited and batch-mode DEP-FFF proved to be 20 times too slow for isolating CTCs from clinical specimens even though cell discrimination and throughput were greatly improved compared with DEP trapping on microarrays.

To meet the high throughput demands for CTC analysis as presented in Table 1, we therefore introduced a variant of batch-mode DEP-FFF called continuous flow DEP-FFF [30,31,128] that retains the advantages of balancing DEP, sedimentation and hydrodynamic lift forces over an extended time to control the vertical position of cells in a laminar flow profile yet no longer exploits cell transport velocity. Instead, using carefully designed slots in the chamber floor to exploit the properties of lamina flow profiles, cells are injected continuously through the chamber floor and flow through conductivity-adjustment and DEP manipulation regions of the chamber where they attain equilibrium heights. The target cancer cells, which reach equilibrium heights close the chamber floor, are then skimmed off through a slot in the chamber bottom. The unwanted blood cells, which reach much higher equilibrium heights under the force balance conditions, remain in the portion of the flow steam that passes over this downstream collection slot and flow to waste. The discriminating power of this method is lower than batch mode DEP-FFF because of limitations in slot performance resulting in imperfections in the skim height. Nevertheless, the DEP crossover frequencies of cancer cells and blood cell subpopulations are sufficiently different that this results in only a small loss of cancer cell isolation efficiency. Most significantly in this arrangement, the specimen is fed in, and CTCs are skimmed off, continuously, allowing a throughput that exceeds 10^6^ nucleated cells·min^−1^ for a 25 mm wide channel while, at the same time, maintaining adequate cell spacing to avoid cell dipole-dipole interactions [122,123]. This approach enables continuous processing of the nucleated cells from a 10 mL clinical blood specimen in 40 min using a 25 mm wide channel. If required, the processing can continue as long as the cells in the specimen are viable and could be implemented in parallel channels, allowing larger volumes of blood to be processed.

**Table 1:** Parameter requirements for isolating cancer cells by DEP for clinical applications.

Parameter	Practical Requirement	Comments
Specimen condition	Cells must have intact membrane barrier function	Membrane barrier function must be intact for DEP to discriminate between cells based on their plasma membrane morphology. Also, cells undergo large dielectric changes in early apoptosis, so stressed specimens are undesirable.
Specimen volume	7 to 10 mL	CTCs are so rare that an accepted working volume to allow for meaningful analysis is around 10 mL
Processing time	<60 min	It is generally accepted that an instrument to make analysis of CTCs a widespread, routine, clinically-relevant procedure needs to be able to process at least one specimen an hour.
Cell throughput	≥10^6^ cells·min^-1^	Even with pre-processing of specimens to remove erythrocytes by lysis or density-gradient separation, a 10 mL specimen contains ~4 × 10^7^ nucleated cells, requiring a high throughput rate to achieve processing of a 10 mL specimen within 60 min.
Suspending medium conductivity	<500 mS·m^−1^ and usually <100 mS·m^−1^	To exploit both positive and negative DEP for cell discrimination, the suspending medium must be of much lower conductivity than the cell cytoplasm, which is ~1,400 mS·m^−1^ due to its physiological ion concentration. The electric current and Joule heating caused by the DEP signal also depends on the suspending medium conductivity.
Suspending medium osmolarity	>200 mOs·kg^−1^ and usually ~300 mOs·kg^−1^	Usually DEP is applied in a suspending medium of low ionic conductivity in which physiological osmolarity (~300 mOs·kg^−1^) is maintained with a non-conductive osmolyte such as sucrose or mannitol. However, osmolarity could be modified substantially to alter cell DEP properties as long as it did not damage the target cancer cell membrane integrity through osmotic stress.
DEP frequency	f > 15 kHz	The DEP frequency is chosen to impose differential forces on the cell types to be separated in accordance with the cell crossover frequencies at the chosen suspending medium conductivity. At low frequencies, charge injection from electrodes becomes greater, increasing the production of electrochemical species that can damage cells. At a conductivity of 30 mS·m^−1^, cells can be protected from such damage for f > 15 kHz by inclusion of catalase in the suspending medium.
Electric field strength	<5 × 10^5^ V·m^−1^ in the highest field regions to which cancer cells are exposed	*F_DEP_* increase with the square of the applied voltage making larger voltages desirable for increased cell separation. However, a transmembrane voltage that may be as large as the product of the cell radius and the local field strength is induced in cells undergoing DEP manipulation. If the applied DEP voltage is too high for large cancer cells attracted to high field regions at electrode edges, the transmembrane potential could induce ion leakage, electroporation, or even electrodestruction, leading to their loss. These problems are averted completely at lower voltages that can still provide good cell separation.
Cell residency time	<400 s	Once cells are suspended in a low conductivity medium, their internal ions begin to leak out, causing their DEP properties to change with time. Tumor cells tend to be leakier than normal cells and their exposure to high field regions during DEP manipulation can induce still faster ion leakage (see Electric Field above). Cell residency time should be short to avert complications caused by changing cell DEP properties.

To assure that the cell properties remain consistent throughout the 40 min processing period for a 10 mL specimen and are not impacted by cytoplasmic ion leakage by being presuspended in a low conductivity DEP buffer, cells are maintained in physiological buffer prior to injection. Their conductivity is adjusted after they have been injected into the DEP chamber just prior to DEP manipulation. To achieve this [30,31,128], cells in physiological buffer are injected through the inlet slot and flow along the chamber floor in a 25 µm thick layer beneath a 275 µm thick layer of sucrose solution that is injected continuously from above and acts as the eluate. Because of the laminar flow characteristics of the chamber, the two fluid layers remain discretely stacked. Ion and sucrose diffusion occur across the interface between these layers and, as the fluid stack travels through the chamber, the conductivity and osmolarity of the two layers equilibrate. Cells, which are too massive to diffuse, remain in the thin specimen layer close to the chamber floor ready for DEP manipulation. Downstream, having attained an equilibrium conductivity of 60 mS·m^−1^ and an osmolarity of 315 mOs·kg^−1^, the cell suspension flows over an array of DEP electrodes. Cells move to equilibrium heights under the influence of DEP, sedimentation and hydrodynamic lift forces as they flow over the microelectrode array. After reaching equilibrium heights, the target cancer cells are skimmed off and blood cells flow to waste. The equilibration of conductivity and osmolarity between the specimen and eluate layers takes less than 200 s and, depending on the cell type, the exposure time to *F_DEP_* ranges from 80 to 300 s. It follows that this approach ensures that all cells from the specimen are exposed to low conductivity medium for the same amount of time, and exhibit uniform DEP responses regardless of when they are injected into the chamber during specimen processing, and that the exposure to DEP signals is short enough to avoid problems associated with cytoplasmic ion leakage. Nevertheless, the same caution about DEP voltage given above for DEP trapping also applies to DEP-FFF. In CTC isolation, the tumor cells are pulled towards the chamber floor where they can be skimmed off while blood cells are levitated above the collection slot. The levitation height of the blood cells increases as the DEP voltage is raised, allowing more latitude in setting the skimming height. However, the CTCs close to the DEP electrodes can experience induced ion leakage or even electroporation or electrodestruction if the voltage is too high. Such undesirable effects result in lower CTC collection efficiency and in reduced viability of the CTCs. Nevertheless, under appropriate operating conditions, the viability of recovered cells is over 90% judged both by trypan blue dye exclusion and by growth experiments in culture.

### 3.4. Collection Purity

The crossover frequencies of the most abundant subpopulations of blood cells, namely lymphocytes and granulocytes, have small standard deviations based on DEP measurements and are separated by at least five and up to seven standard deviations from the crossover frequencies of most cancer cells, suggesting that cancer cells should be separable from blood with high efficiency and purity [28,29]. We conducted studies with several cancer cell lines including MDA-MB-435, MDA-MB-231 spiked into 2 × 10^7^ PBMNs. Cancer cells could be recovered with efficiencies ranging from 40%–50% by DEP trapping (by flowing PBMNs through a 25 mm wide × 300 mm long DEP microelectrode over a 30 min period and then turning off the DEP field to release the trapped cells). Batch mode DEP-FFF allowed cancer cell recovery up to 95% efficiency in an elution peak emerging 450–700 s after each run was started (blood cells eluted at 150–300 s). However, the cell batch size had to be limited to attain high efficiency [23]. Continuous-flow DEP-FFF provided a cancer cell recovery efficiency of 70%–85% and processed 10^6^ cells·min^−1^ [23,30,31,78]. In all cases, the recovered cells were viable as judged by trypan blue staining and they could be cultured normally. We examined the molecular characteristics of MDA-MB-231 cells recovered from PBMNs by batch mode DEP-FFF and they showed unaltered genomic profiles [30]. Furthermore, changes in gene expression of these cells after DEP-FFF processing were found to be no greater than gene expression changes caused by exposure to PBMNs and switching the cells between growth media during manipulations [30]. This suggests that, while DEP did not *exacerbate* changes in gene expression, great caution is needed when trying to correlate gene expression profiles of CTCs, isolated by any method, with those in the tumors of origin. Evidently, gene expression is extremely labile and mirrors the state of the cell at the time it is analyzed.

Isolation efficiency of the cancer cells was independent of cell surface markers such as EpCAM and growth receptors in all DEP methods and EpCAM-negative as well as “triple negative” breast cancer lines were collected with the same efficiency as EpCAM-positive and receptor-positive types [23]. This is expected for DEP and any other isolation method that depends upon cell physical properties.

Cell purity of the cancer cell isolates was also examined. In general, this was far lower than we expected based on the statistical distributions measured for the cell dielectric properties, particularly for continuous-flow DEP-FFF. We found that this resulted from the presence of damaged PBMNs in our cancer cell isolates. This could be explained by the life span of PBMNs in the blood. A 10 mL clinical specimen contains around 4 × 10^7^ PBMNs of which approximately 0.1%, or ~4 × 10^4^ cells, are close enough to the end of their lifespan that their membrane integrity is weak. These cells leak cytoplasmic ions in the low conductivity DEP buffer. Instead of deflecting the electric field and experiencing DEP levitation (Figure 1B), the field passes straight through them (Figure 1C), they do not exhibit DEP responses, and they sediment to the floor of the DEP-FFF chamber where they are skimmed off with the target cancer cells. This leads to anomalously low recovery purity because the target cancer cells are collected against a background of ~4 × 10^4^ senescent peripheral blood cells. Recovery of viable CTCs from these non-viable PBMNs by a small additional DEP stage would be straightforward if higher isolation purity is required. Tests on normal blood spiked with cultured MDA-MB-231 cells using a secondary DEP stage to process emerging cells showed that cancer cell purities as high as 20% could be achieved. Parenthetically, these observations highlight that unless specimens can be fixed by some method that seals their membranes against ions leakage, specimen collection, transport and preparation steps used in DEP isolation of CTCs need to avoid loss of cell viability.

### 3.5. Cancer Cell Isolation Findings

In collaboration with Dr. Apostolia Tsimberidou (The University of Texas M.D. Anderson Cancer Center, Houston, TX, USA), 45 blood specimens of patients recently diagnosed with late stage colon, liver, colorectal or colon-liver cancers were analyzed to demonstrate that CTCs could be isolated from blood under realistic clinical conditions. Peripheral blood specimens were obtained as part of the Initiative for Molecular Profiling in Advanced Cancer Therapy (IMPACT) Trial at The University of Texas M.D. Anderson Cancer Center with informed patient consent and the approval of Institutional Biosafety Committee. These were collected and processed as described previously [30]. Based on immunostaining for cytokeratin using FITC-conjugated CK3-6H5 antibodies (Miltenyi Biotec, Bergisch Gladbach, Germany) combined with nuclear staining by DAPI (D1306 Molecular Probes, Eugene, OR, USA), CTCs were isolated from all specimens. Ten specimens were split into stained and unstained aliquots. Stained aliquots were subjected to analysis for CTCs by fluorescence microscopy. Unstained cells were transferred into culture in a petri dish containing 2 mL RPMI media supplemented with 20% fetal bovine serum and Pen/Strep antibiotic at 37 °C under a 95% air/ 5% CO2 atmosphere. In tests lasting as long as 60 days with weekly replacement of media, larger cells, putatively identified as CTCs, remained alive but did not undergo more than one or two divisions. This suggests that the isolated CTCs were viable and had growth potential but that more sophisticated methods are needed if cultures suitable for drug testing and other studies are to be established.

Although comparison studies using a currently accepted benchmark such as CellSearch analysis were not conducted by us on these clinical specimens, our results confirmed that continuous-flow DEP-FFF isolated CTCs from clinical specimens. Comprehensive comparison studies with several CTC isolation methods are being conducted by ApoCell [129], the licensee of the technology. A number of results demonstrating the isolation of CTCs by DEP-FFF from breast, ovary, colon, lung and liver specimens, including EpCAM-negative and other challenging specimens have been presented [130,131,132]. These results show that the DEP-FFF method appears to capture more CTCs than the CellSearch method in many cases, including cells of EpCAM negative and other variant phenotypes.

### 3.6. Post Processing

CTCs isolated from specimens by DEP methods such as DEP-FFF emerge in a flow stream at a flow rate of 25 µL·min^−1^ and may be processed by additional microfluidic stages. These could incorporate additional sorting steps based on high-discrimination DEP methods that are better suited to low-throughput or other chip-based analyses. For example, single CTCs could be sorted or subjected to more sophisticated analysis to provide tumor heterogeneity information including lab-on-chip shotgun PCR analysis of gene targets. As a visionary example of the technical possibilities afforded by the microfluidic output of DEP-FFF, most molecular analysis platforms now employ microfluidic cores that could, in principle, be directly interfaced to DEP-FFF via a microfluidic interface. If CTCs fulfill their promise for enhanced routine prognosis and diagnosis, instruments can be envisioned in which blood specimens are processed from tube to molecular data to provide details of tumor profiles and heterogeneity from a single instrument without operator intervention. DEP-FFF stages could provide integrated front ends for routine comprehensive analysis of blood specimens.

## 4. Conclusions

Dielectrophoresis allows the intrinsic dielectric properties of suspended cells to be characterized and exploited for cell discrimination and separation. Consistent dielectric differences have been observed between transformed and comparable normal cells and between all types of cancer cells that have been studied and the normal cell subpopulations of peripheral blood. This article summarizes the principles of DEP for discriminating between and isolating cancer, normal and blood cell types and considers the biological basis for the observed differences in the dielectric characteristics of the cells. Cell membrane area emerges as a cell morphological characteristic that reflects the organization of the tissue from which the cells are derived and determines the dielectric properties of the cells in suspension. Because increasing disorder is a hallmark of cancer, cell dielectric properties can be expected to reflect cancer progression regardless of the tumor of origin. Therefore, DEP methods appear likely to be applicable to the isolation of cells from all types of solid tumors and also applicable to highly concentrating leukemia cells.

The current generation of continuous-flow DEP-FFF devices can sort in excess of 10^6^ nucleated cells·min^−1^ and can isolate CTCs from 10 mL clinical blood specimens in less than 1 h. The isolation of CTCs through all DEP methods is independent of cell surface markers and the isolated cells are viable and can be maintained in culture. So far, clinical studies confirm that DEP is an antibody-independent isolation method permitting the capture of CTCs regardless of their surface marker profiles. In trials so far CTC capture rates by DEP-FFF often exceed those of other methods.

## Appendix

### A1. Dielectrophoresis of Mammalian Cells

In a stationary, inhomogeneous AC electric field  (*f*) [62,67,122,133], the DEP force *F_DEP_* acting on a cell can be written as:

*F_DEP_* = 2*πε_s_**R*^3^*f_CM_*(*f*).▽*E_RMS_*^2^ (A1)

(A2)

is the real part of the so-called Clausius-Mossotti factor, *R* is the cell radius, *f* is the frequency of the applied electric field, *E_RMS_* is its RMS intensity, and the electric field inhomogeneity is expressed by the ▽*E_RMS_*^2^ term, which reflects the geometry of the DEP electrodes. The Clausius-Mossotti factor embodies the dielectric properties of the cell in suspension through an effective frequency:

(A3)

where *ε_p_* and *ε_s_* are the real permittvities (dielectric constants) and *σ_p_* and *σ_s_* are the electrical conductivities of the cell and its suspending medium, respectively [133]. For viable cells that are suspended in a low conductivity medium, *σ_s_* ˃ *σ_p_* and *ε_p_* ˃ *ε_s_* and *f*_o_ is real. In this case, there is a value of the frequency *f* = *f*_o_ in Equation (A.2) at which the real part of the Claussius-Mossotti factor becomes zero and cells experience no *F_DEP_*. Depending on whether the applied frequency *f* is above or below *f*_o_, the *F_DEP_* will move particles towards strong or weak field regions, respectively. Because the *F_DEP_* changes direction at *f*_o_, this frequency is called *the DEP crossover frequency* [133].

The electrical properties of most mammalian cell types can be related to cell structure and composition by the single shell dielectric model [67,134,135], in which a cell is idealized as a homogeneous spherical core of radius *R*, representing the cytoplasm of complex permittivity  = *ε_i_* − *jσ_i_*/(2π *f*), surrounded by a thin shell of thickness *d*, representing the membrane of complex permittivity  = *ε_mem_* − *jσ_mem_*/(2π *f*). The single shell model gives the complex permittivity of the cell [67,134,135] as:

(A4)

If we assume the cell membrane is a lipid bilayer and that the aqueous cytoplasm has physiological ionic conductivity then σ_i_ ˃˃ σ_mem_ and ε_i_ ˃˃ ε_mem_, so that  ˃˃  and, for frequencies between approximately 10 kHz and 1 MHz [67,136], this may be approximated as:

(A5)

which, in turn, allows the specific capacitance and specific conductance of the cell membrane [67,136] to be approximated as:

(A6)

respectively. Then the cell crossover frequency in Equation (A.3) can be expressed as:

(A7)

If the cell membrane barrier function breaks down then *R.G_mem_* ˃˃ *σ*_s_ and there is not a real solution for *f*_o_, meaning that the cell will not exhibit a DEP crossover frequency. Additionally, poor cell membrane barrier function associated with intermediate and large values of *G_mem_* will allow cytoplasmic ions to leak from the cell allowing the conductivities of the cytoplasm and suspending medium to equilibrate quickly during experiments and the *F_DEP_* will vanish. Consequently, non-viable cells experience neither repulsive nor attractive *F_DEP_* and are effectively invisible to the electric field. From an experimental point of view, they settle in a DEP chamber as inert particles.

On the other hand, viable cells have an intact membrane barrier function with a very low conductivity, so that *R.G_mem_* ˂˂ *σ_s_*. Therefore cytoplasmic conductivity will remain high compared with the suspending medium. Because *R.C_mem_* ˃˃ *ε*_s_ for all mammalian cells, viable cells will exhibit a crossover frequency that can be approximated from Equation (A.7) as [67]:

(A8)

Here, the crossover frequency is real and the cells exhibit positive or negative *F_DEP_* according to whether the applied frequency is above or below it. Note that this expression is not valid for very small cells such as bacteria because effects associated with conductivity around the cells, which becomes important at smaller size scales, has been ignored. By constructing synthetic vesicles of various structures and examining their electrokinetic properties, we provided experimental validation for the application of the shell model to deriving dielectric properties of the membranes and interior of cell-like structures [67].

### A2. Cell Morphological Considerations

Smooth cell plasma membrane has a capacitance of *C*_0_ ≈ 9 mF·m^−2^ [137]. However, the surfaces of cells are wrinkled with microvilli, ruffles and folds. These features increase the area of membrane that covers the cell compared to the area of perfectly smooth sphere. It follows that the membrane capacitance of any cell may be written as *C_mem _*= *ϕC*_0_, where the factor *ϕ* ≥ 1 takes into account the extra cell membrane surface area due to “wrinkling”. Electron microscopy has confirmed that ruffles, folds and microvilli play an important role in determining cell capacitance and crossover frequency [67,78]. It follows, then from Equation (A.8) that two cellular properties determine the crossover frequency, namely the cell radius *R* and the membrane folding factor *ϕ*. The product of these two parameters (*R∙ϕ*) determines the “dielectric phenotype” of a given cell type at any given suspending medium conductivity *σ_s_*, distinguishing DEP methods from size-filtering approaches to cell isolation that depend on size alone:

(A9)

Cell membrane folding occurs on many size scales and we have conducted a more sophisticated dielectric analysis that accounts for this through a fractal approach [68]. This yields a somewhat different expression for the relationship between cell surface morphology and dielectric behavior yet the result is qualitatively and philosophically in agreement with the simple folding model presented here, which we consider adequate for the purpose of understanding cell isolation. While CTCs often have larger radii than PBMNs, some CTCs are as small as PBMNs, showing that cell radius alone cannot be used as a reliable basis for isolating CTCs. Therefore it is of interest to investigate whether there are differences between the folding factors of CTCs and blood cells that would cause crossover frequencies of CTCs to be consistently different from PBMNs. Recently, we showed that the folding factors *ϕ* of forty types of cultured cells reflected their morphological properties prior to harvest [29]. This result can be understood in terms of conservation of cell integrity of viable cells during harvest. When it is part of a tissue, a cell’s morphology conforms to the cells and structures around it and it has a membrane area *A* and volume *V* that are consistent with this configuration. Upon release from the tissue, the cell loses the anchorages that maintained its local conformation and its cytoskeleton causes it to round up into an essentially spherical shape. Assuming the cell remains intact during this process, the cytoplasmic volume and cell membrane area will be conserved. To conserve volume, the cell will assume a radius:

(A10)

Meanwhile, its membrane area *A,* which previously conformed to neighboring cells and structure in the tissue, now has to be accommodated on the surface of the newly rounded cell. Consequently, it will need to assume a membrane folding factor:

*ϕ* = A/(4*π*∙*R*^2^) (A11)

Combining these expressions, we find:

(A12)

By combining this with expression 4.2 for crossover frequency, we obtain [29]:

(A13)

This expression relates the cell crossover frequency to cell morphology and reveals the biological basis of the cell dielectric properties that pertain to cell discrimination by DEP.

### A3. Separability of Cells by DEP Trapping

If, in a cell mixture, one cell type is to be trapped by DEP against the Stokes drag of a fluid flow stream while another cell type is to be swept away, we have shown previously that there is a limit to how well the cell types can be discriminated. The efficiency of separation is related to the separability factor given by:

*S* = *R*_1_*f*_CM1_(*f*) − *R*_2_*f*_CM2_(*f*) (A14)

where *f*_CM1_(*f*) and *f*_CM2_(*f*) are the real parts of the Clausius-Mossotti factors and *R*_1_ and *R*_2_ are the radii of the two cell types, respectively [79,81].

Using differential calculus, we showed [79] that the maximum separability of the two cell types is:

(A15)

at a frequency:

(A16)

where:

(A17)

The cell type that is trapped, which in this case is the species having radius *R*_2_ and membrane folding factor *ϕ*_2_, will be subjected to a *F_DEP_* for trapping of:

(A18)

Voldman has also considered and modeled the case in which DEP trapping may be overcome by fluid flow forces for a particular trapping configuration [119]. However, these models do not take into account thermal agitation effects. If we assume that an effective displacement *D*_2_ is sufficient to destabilize a cell so that it leaves a DEP trap, we can write the critical trapping energy as *F_DEP_*_2_∙*D*_2_. The frequency with which Brownian motion will provide sufficient destabilizing energy to de-trap the cell can then be written from conventional transition state theory based on the approach of Arrhenius as:

(A19)

where *k_B_* is the Boltzmann constant, *T* is the absolute temperature, and *h* is Planck’s constant. This gives the mean escape time from trapping due to thermal effects as  = *k*^−1^. In our experiments,  can be as long as thousands of seconds when the trapping is strong or fractions of a second when the trapping is weak [56]. Of course, the trapping may be extended indefinitely by using methods to adhere cells to the DEP trapping sites, if desired [113,138].

Two key principles emerge from this analysis of DEP trapping, namely that both the ability to discriminate between cells by DEP trapping and discrete deflection and the mean time for which cells can be trapped before thermal effects shake them free both diminish as the dielectric difference between the different cell types decreases. This sets a fundamental limit on the discriminating power of methods that employ single DEP trapping or discrete deflection. This problem can be ameliorated to a considerable degree by using a device with multiple trapping or discrete deflection sites. In such cases, thermal effects are still present and influence cell behavior at each DEP interaction site but these fluctuations tend to be averaged out over hundreds or thousands of capture or deflection events [138,139]. These devices may also be combined with immunoaffinity methods to enhance cell discrimination [140].

### A4. Separation by the Force Equilibrium Methods of DEP-FFF

As an alternative cell separation strategy to confinement by a potential barrier, cells can be discriminated by DEP based on a balance of forces. In this case, a periodic array of DEP microelectrodes can be employed to provide a DEP field that is continuous in the cell manipulation space [125,141]. Employing this approach in the DEP-FFF method, sedimentation, DEP and hydrodynamic lift forces are imposed on cells to control their heights in a Poiseille flow profile as they flow through a separation chamber:

*F_sed_* + *F_DEP_* + *F_HDL_* = 0 (A20)

Because *F_DEP_* does not have discontinuities above the microelectrode array, this balance can be expressed [31,78] in terms of the cell physical properties as a continuous equation:

(A.21)

where *A*, *B*, *C* and *d* are constants for given DEP-FFF operating conditions, Δ*ρ* is the difference between the cell and suspending medium densities, Ф describes the deformability of the cell in the eluate flow profle, and *f*_0_ is the cell crossover frequency as used above. This equation balances at some height *h* according to the cell physical properties. Cells having different properties attain different equilibrium heights and are carried at different speeds, *v*(*h*), by the eluate flow according to the blended expression [78]:

(A22)

where  is the shear rate of the eluate flow profile at the chamber floor. Cells elute from the DEP-FFF microelectrode array after a time *T_elute_*(*h*) = *L*/*v*(*h*), where L is the length of array. If the entire DEP-FFF chamber is lined with DEP microelectrodes then L is the length of the DEP-FFF chamber. Thus different cell types starting off together at the front end of the chamber will elute at different times. This is the principle of batch-mode DEP-FFF.

Alternatively, because different cell types are levitated to different heights, target cells may be differentially skimmed from the flow stream after they have reached their equilibrium heights using a suitable arrangement of outlet ports [30,31,129]. This approach allows for continuous injection and skimming of cells. Both of these DEP-FFF methods average out thermal fluctuations during the separation process and overcome the discrimination limits of the DEP trapping and discrete deflection approaches.
